# Supplementary material for: The Ability of Different Tea Tree Germplasm Resources in South China to Aggregate Rhizosphere Soil Characteristic Fungi Affects Tea Quality
Source: Plants (Basel). 2024 Jul 24;13(15):2029. doi: 10.3390/plants13152029 (PMC11314174; doi:10.3390/plants13152029)
Supplement: Supplementary file 1 [file plants-13-02029-s001.zip › plants-3087822-supplementary.pdf]

Supplementary Information

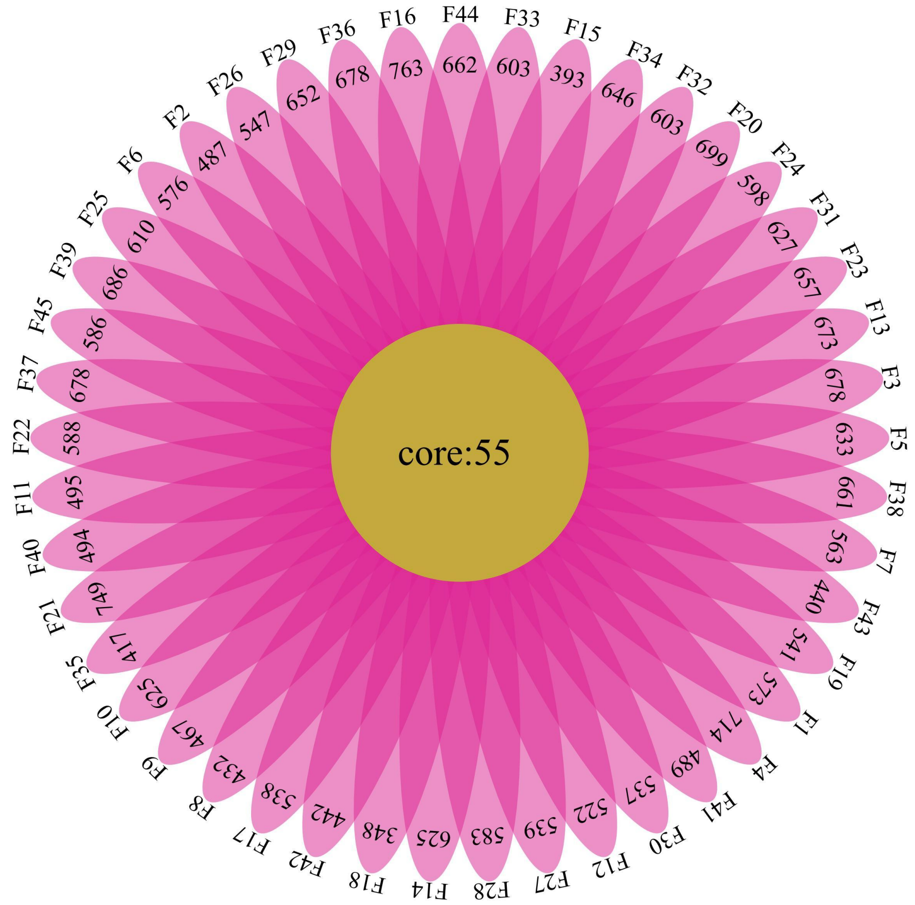

Figure S1. Petalogram analysis of the distribution of OTUs in rhizosphere soils of tea trees. F1~F45 represent the serial numbers of 45 tea tree germplasm resources (Supplementary Table S1). Each petal represents a tea tree variety, the core numbers in the center represent the number of OTUs common to different tea tree varieties, and the numbers on the petals represent the number of OTUs specific to each tea tree variety.

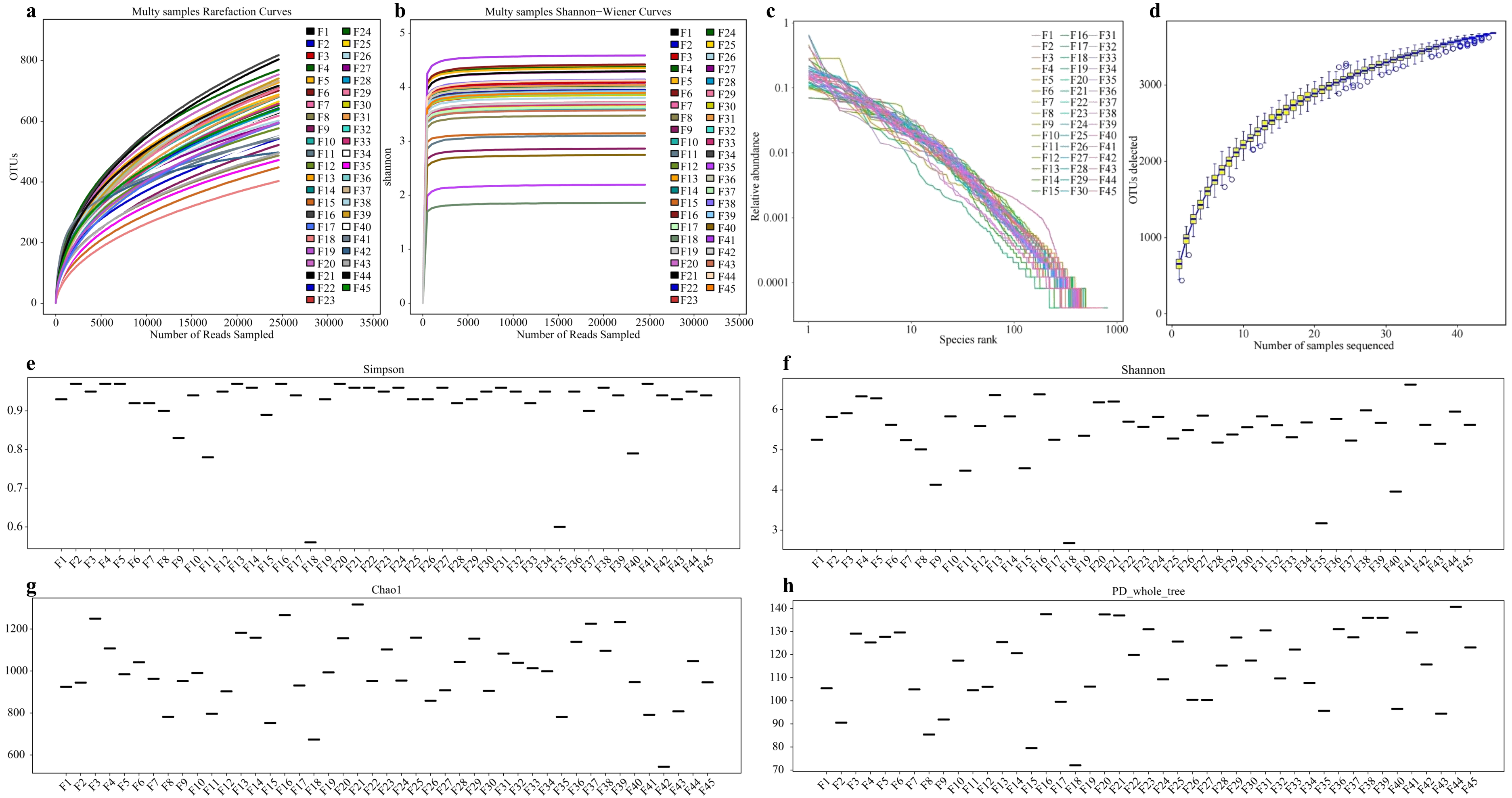

Figure S2. OTUs analysis of rhizosphere soil fungi of tea tree germplasm resources. F1~F45 represent the serial numbers of 45 tea tree germplasm resources (Supplementary Table S1). **a** Analysis of rarefaction curves of fungal OTUs; **b** Analysis of shannon-wiener curves of fungal diversity; **c** Rank-abundance plots of changes in fungal abundance; **d** Species accumulation plots of fungi; **e** Analysis of simpson's indexes of fungi; **f** Analysis of shannon's indexes of fungi; **g** Analysis of Chao1 indexes of fungi; **h** Analysis of PD whole tree indexes of fungi.

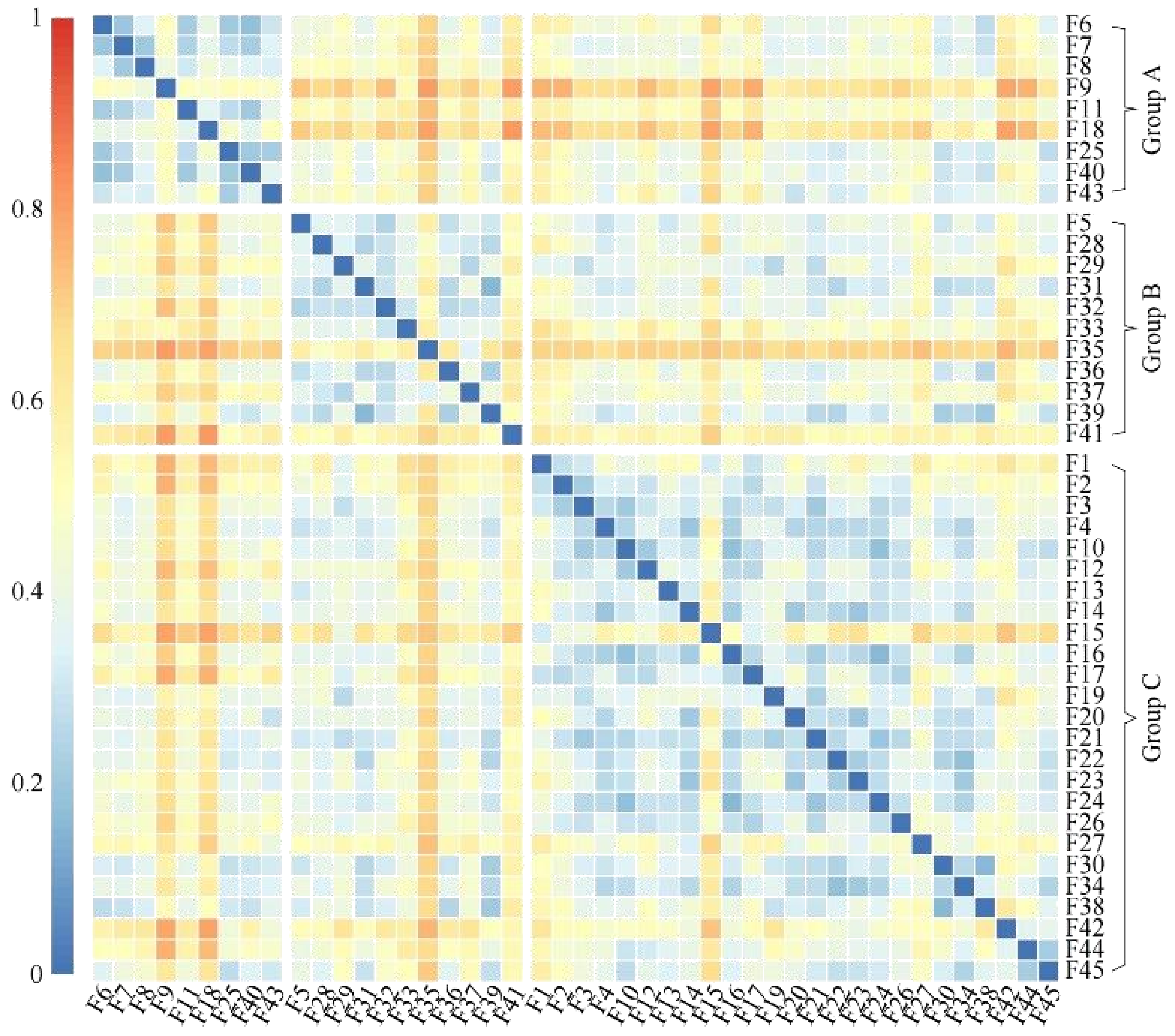

Figure S3. Bray-curtis heat map of rhizosphere soil fungal abundance of tea tree germplasm resources. F1~F45 denote the serial numbers of the 45 tea tree germplasm resources (Supplementary Table S1). Group A, B, C denote the three categories into which the 45 tea tree germplasm resources were classified by unsupervised K-mean clustering (see Supplementary Table S5 for the tea tree varieties corresponding to Group A, B, C).

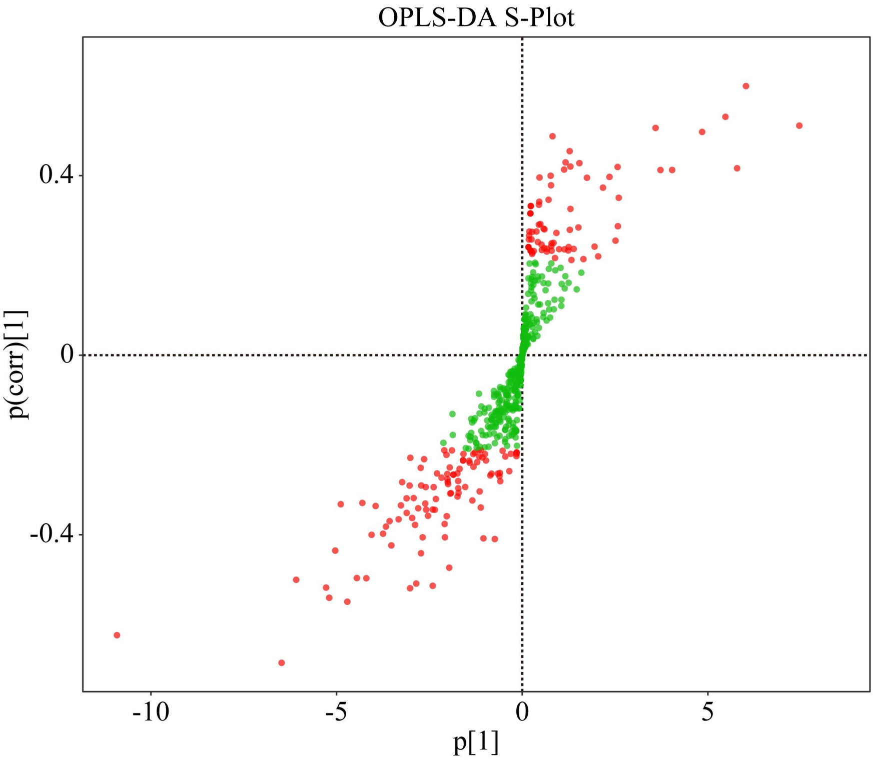

Figure S4. Key fungi with VIP>1 were obtained through OPLS-DA model constructed by soil fungal abundance. Red dots indicate fungi with VIP > 1.

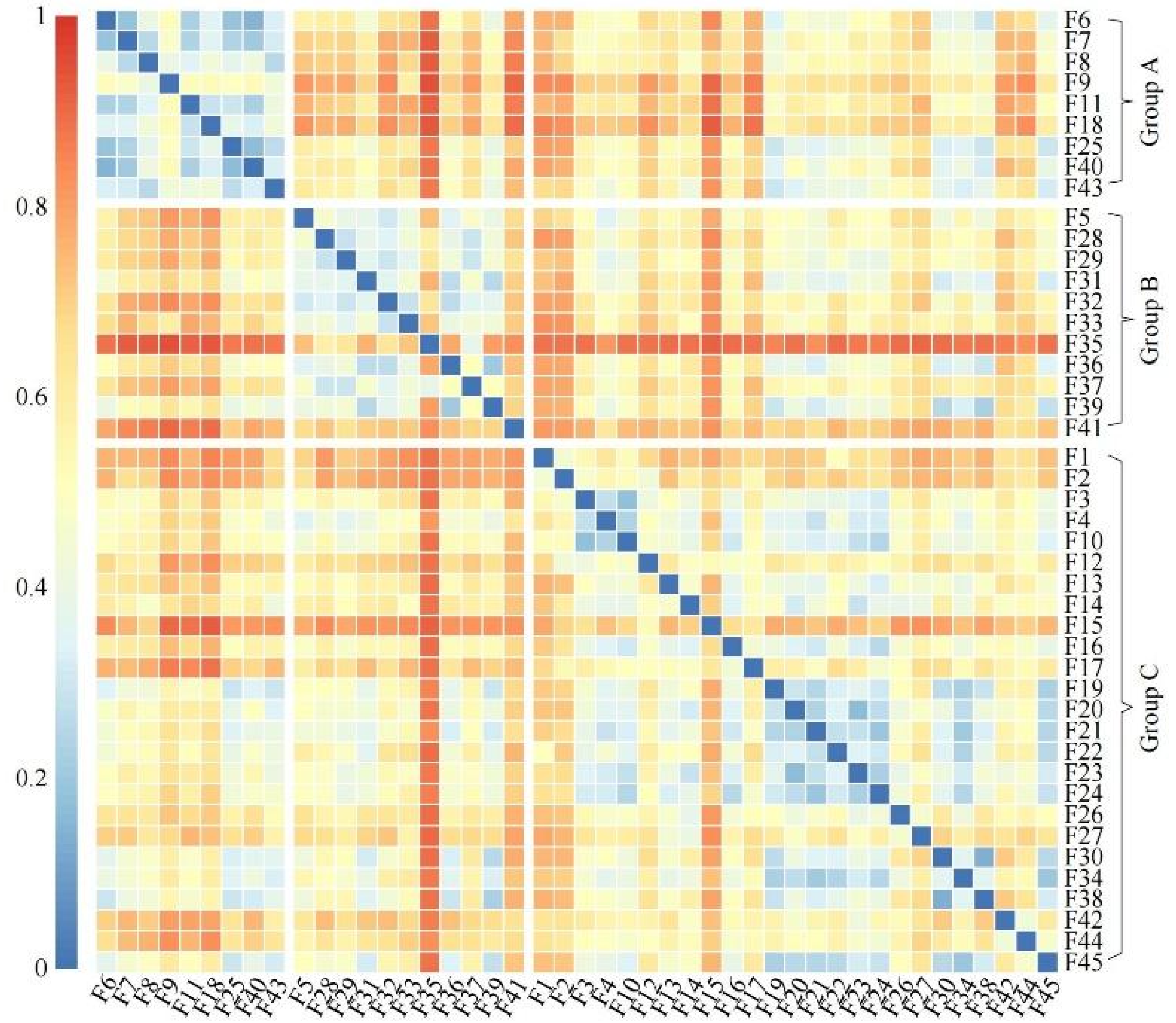

Figure S5. Bray-curtis heat map analysis based on the abundance of 181 key fungal genera. F1~F45 indicate the numbers of 45 tea tree germplasm resources (see Supplementary Table S1 for the tea tree varieties corresponding to the numbers). Group A, B, C denote the three categories into which the 45 tea tree germplasm resources were classified after unsupervised K-mean clustering (see Supplementary Table S5 for the tea tree varieties corresponding to Group A, B, C).

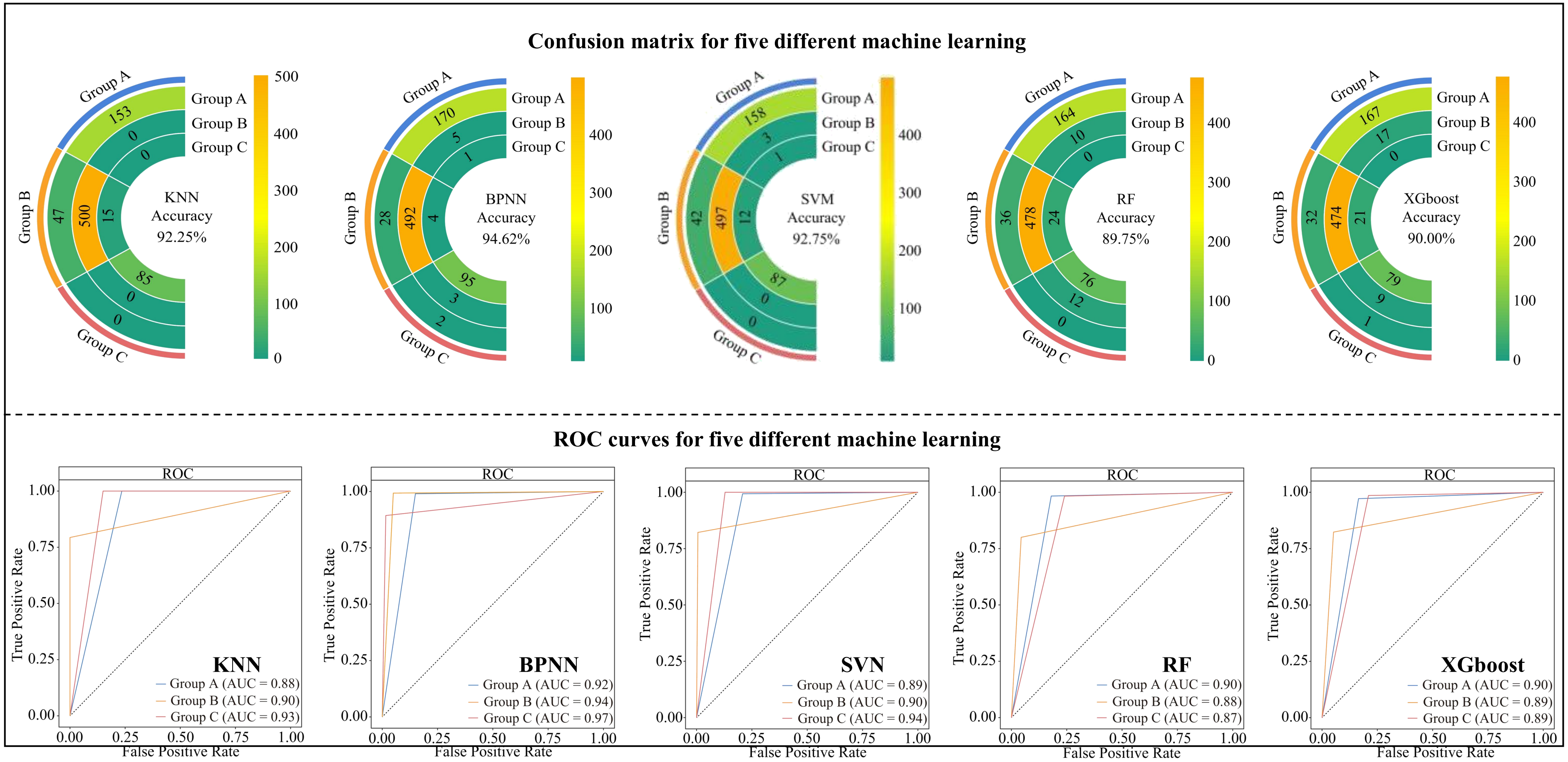

Figure S6. Machine deep learning simulations based on the abundance of 181 key fungal genera to validate classification accuracy. KNN, BPNN, SVM, RF, XGboost denote five types of machine deep learning such as k-nearest neighbor, support vector machine, back propagation neural network, random forest, extreme gradient boosting. Group A, B, C denote the three categories into which the 45 tea tree germplasm resources were classified after unsupervised K-mean clustering (see Supplementary Table S5 for the tea tree varieties corresponding to Group A, B, C).

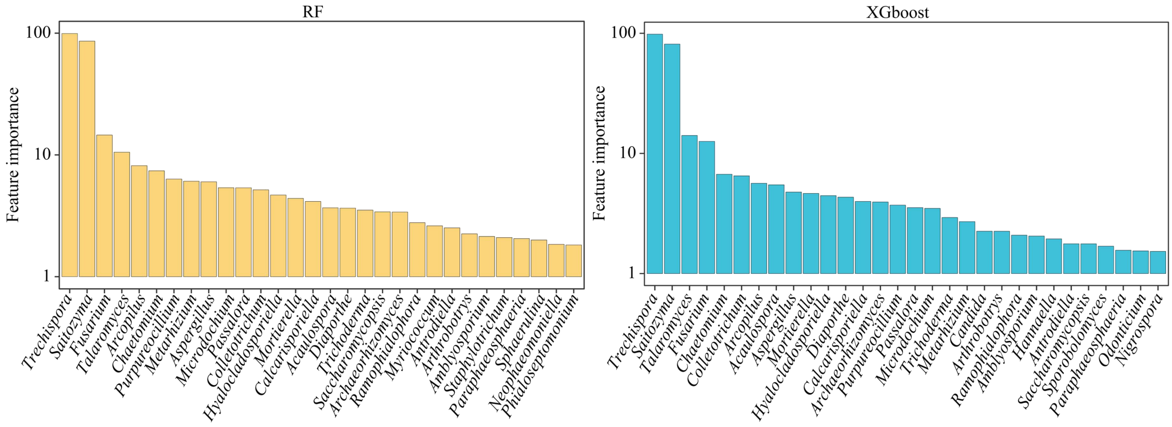

Figure S7. Construction of RF and XGboost machine deep learning models to obtain the top 30 fungal genera in terms of feature importance values

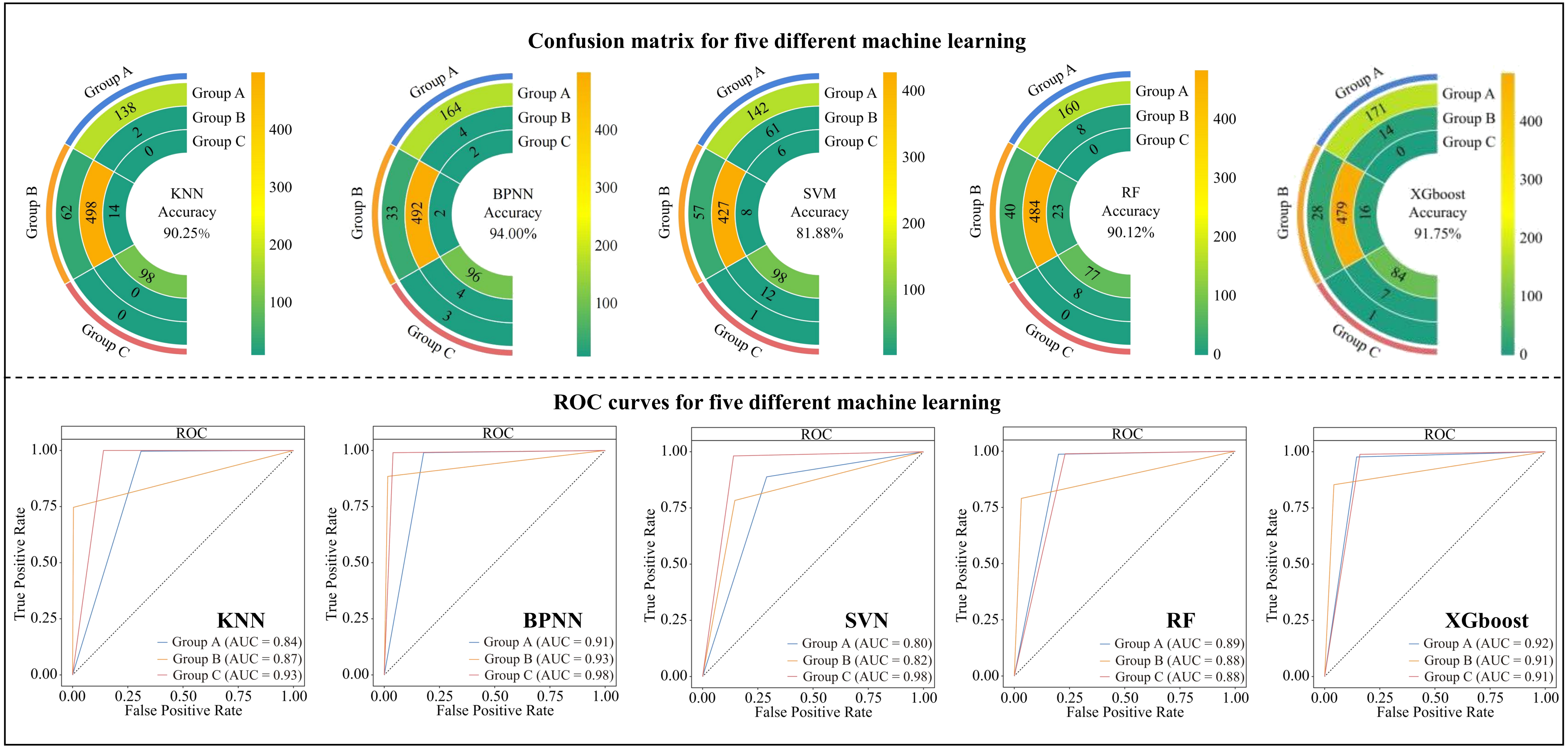

Figure S8. Machine deep learning based on the abundance of 25 characteristic fungal genera to validate classification accuracy. KNN, BPNN, SVM, RF, XGboost denote five types of machine deep learning such as k-nearest neighbor, support vector machine, back propagation neural network, random forest, extreme gradient boosting. Group A, B, C denote the three categories into which the 45 tea tree germplasm resources were classified after unsupervised K-mean clustering (see Supplementary Table S5 for the tea tree varieties corresponding to Group A, B, C).

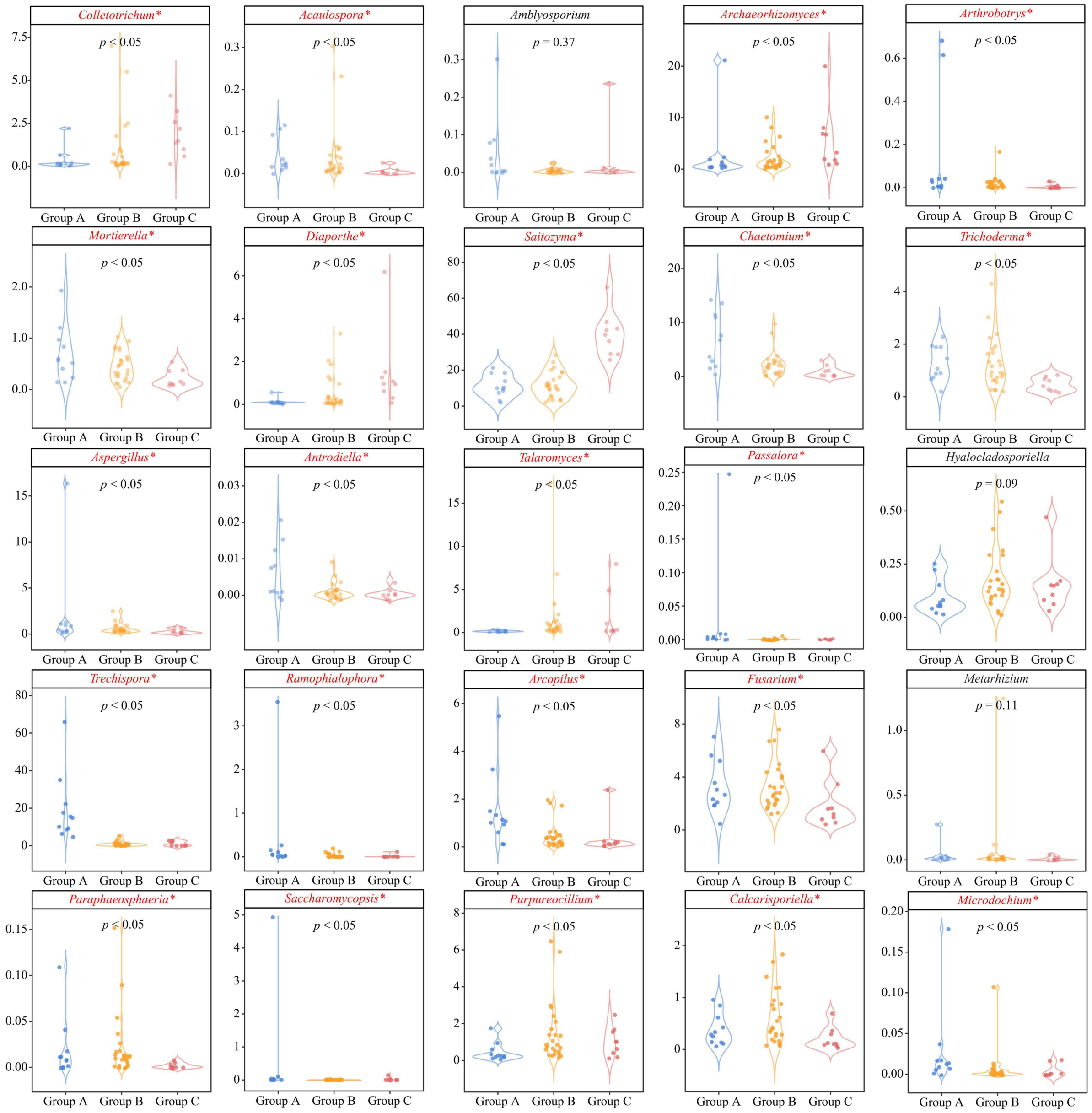

Figure S9. Differential analysis of the abundance of 25 characteristic fungal genera. Group A, B, C denote the three categories into which the 45 tea tree germplasm resources were classified after unsupervised K-mean clustering (see Supplementary Table S5 for the tea tree varieties corresponding to Group A, B, C). *Indicates that the abundance of the fungus differed among groups A, B, and C at the $p < 0.05$ level. Kruskal-Wallis was used to test the significance of differences among the three groups at the $p < 0.05$ level.

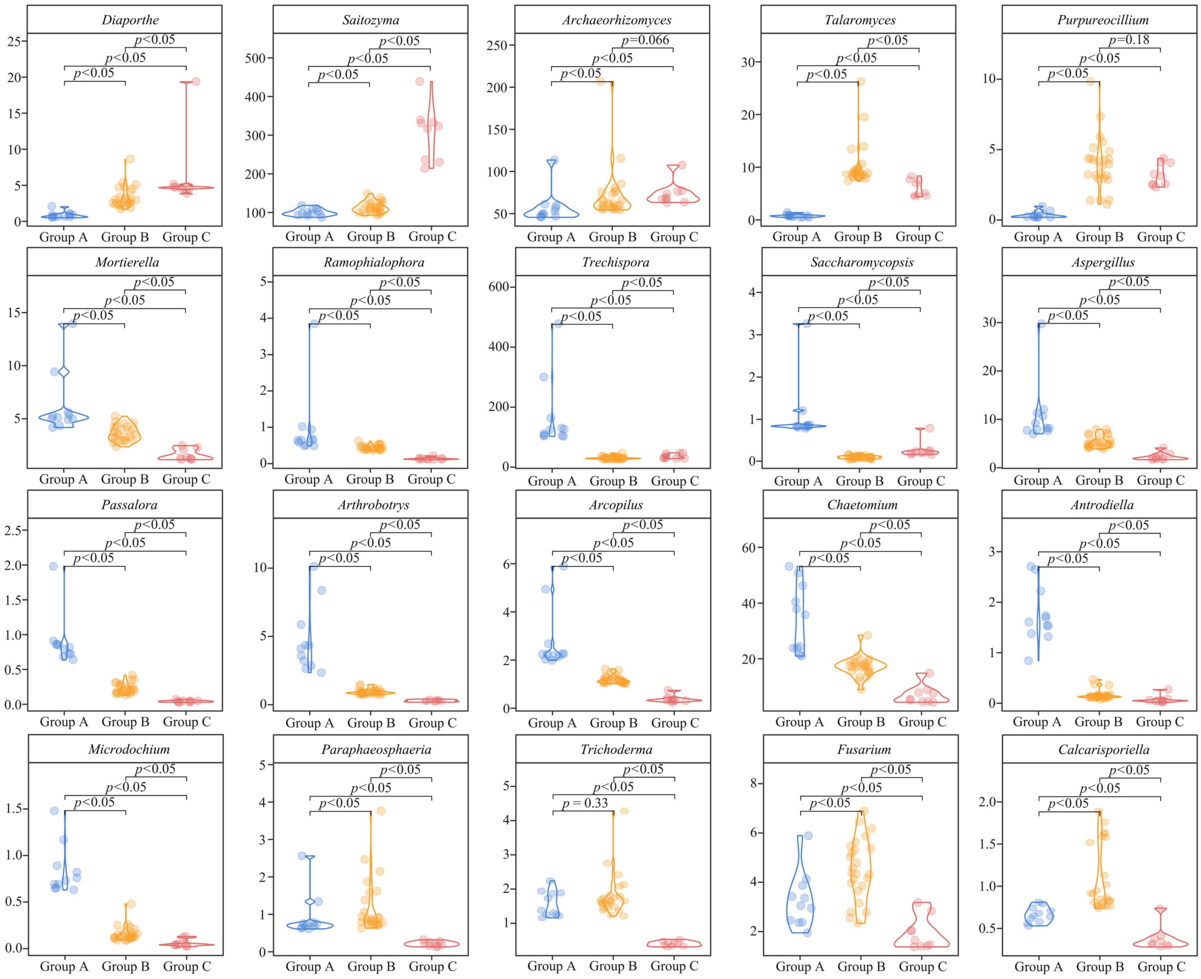

Figure S10. *q*RT-PCR differential analysis of 20 characteristic fungal genera. Group A, B, C denote the three categories into which the 45 tea tree germplasm resources were classified after unsupervised K-mean clustering (see Supplementary Table S5 for the tea tree varieties corresponding to Group A, B, C). Wilcox.test was used to test the significance of differences between the two groups at the $p < 0.05$ level.

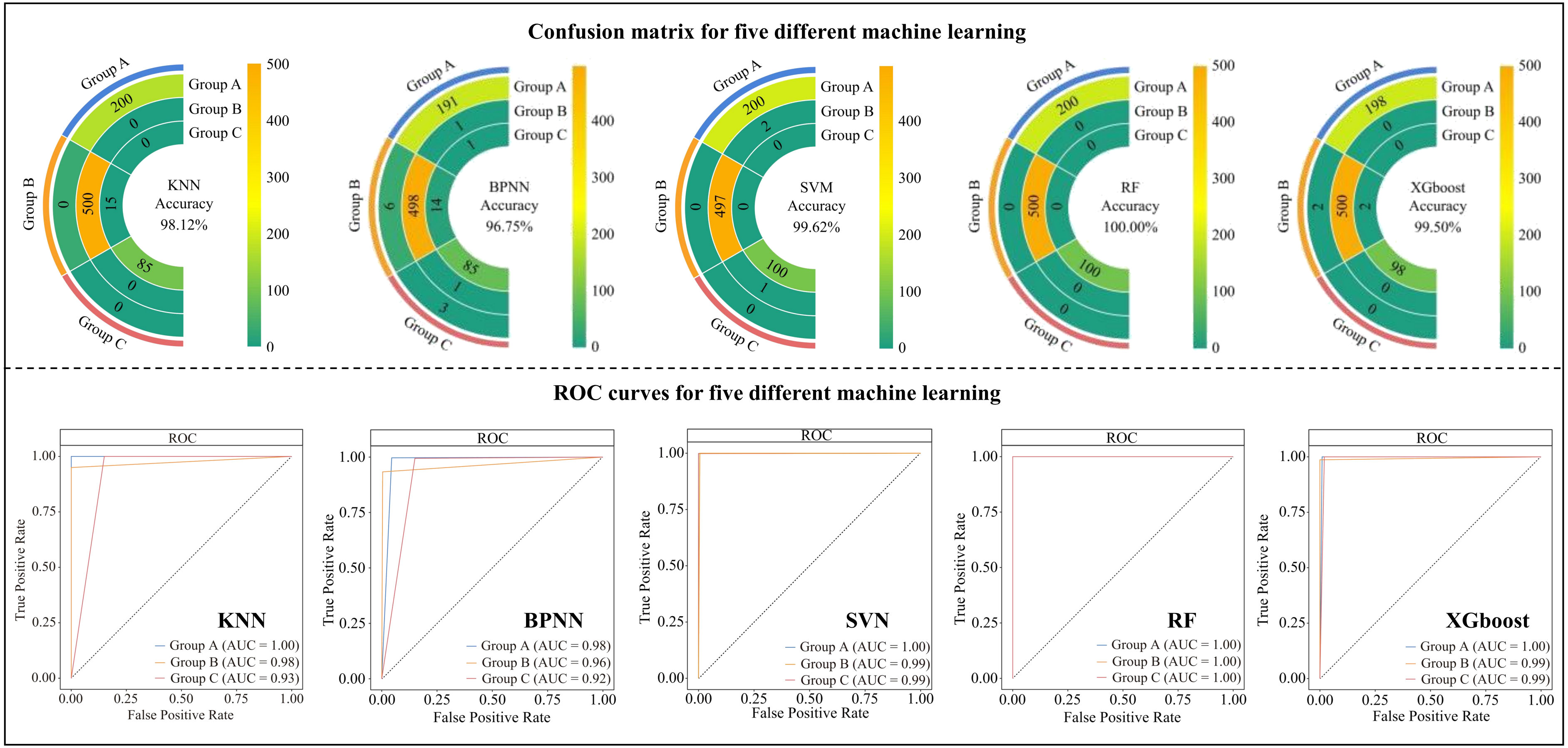

Figure S11. Machine deep learning based on *q*RT-PCR quantification results of 20 characteristic fungal genera to validate classification accuracy. KNN, BPNN, SVN, RF and XGboost represent five machine deep learning such as k-nearest neighbor, back propagation neural network, support vector machine; random forest, eXtreme gradient random forest, eXtreme gradient boosting, respectively. Group A, B, and C represent the three categories into which the 45 tea tree germplasm resources were classified after unsupervised k-mean clustering (see Supplementary Table S5 for the tea tree species corresponding to Group A, B, and C).

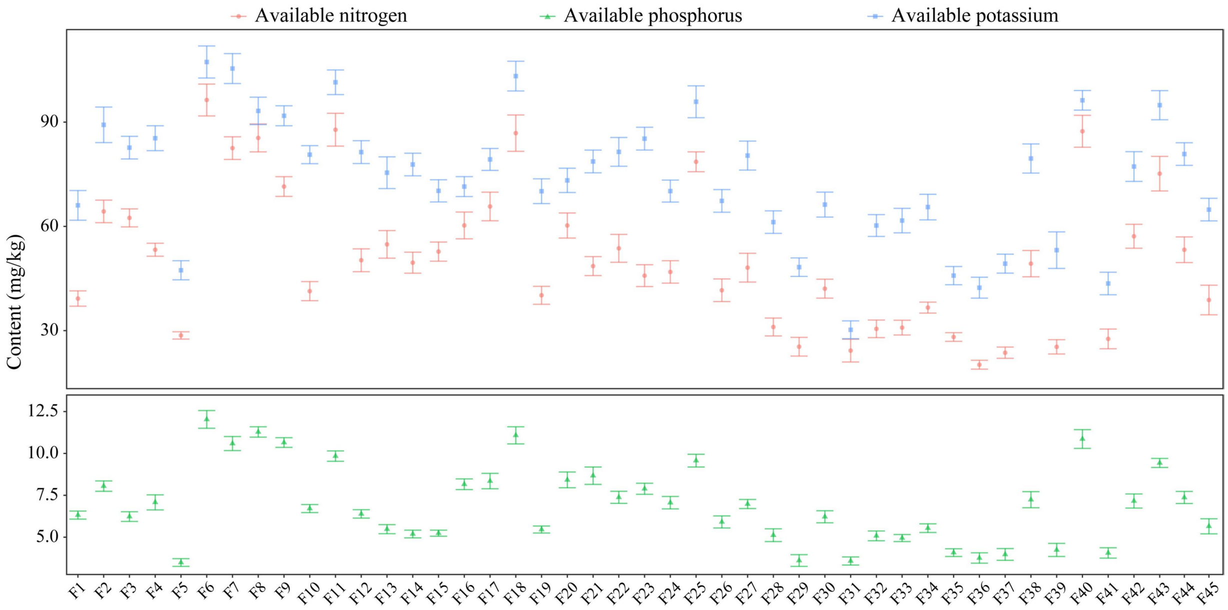

Figure S12. Content of available nitrogen, available phosphorus, available potassium in leaves of 45 tea germplasm resources. Data are presented as means and standard deviations (means ± SD).

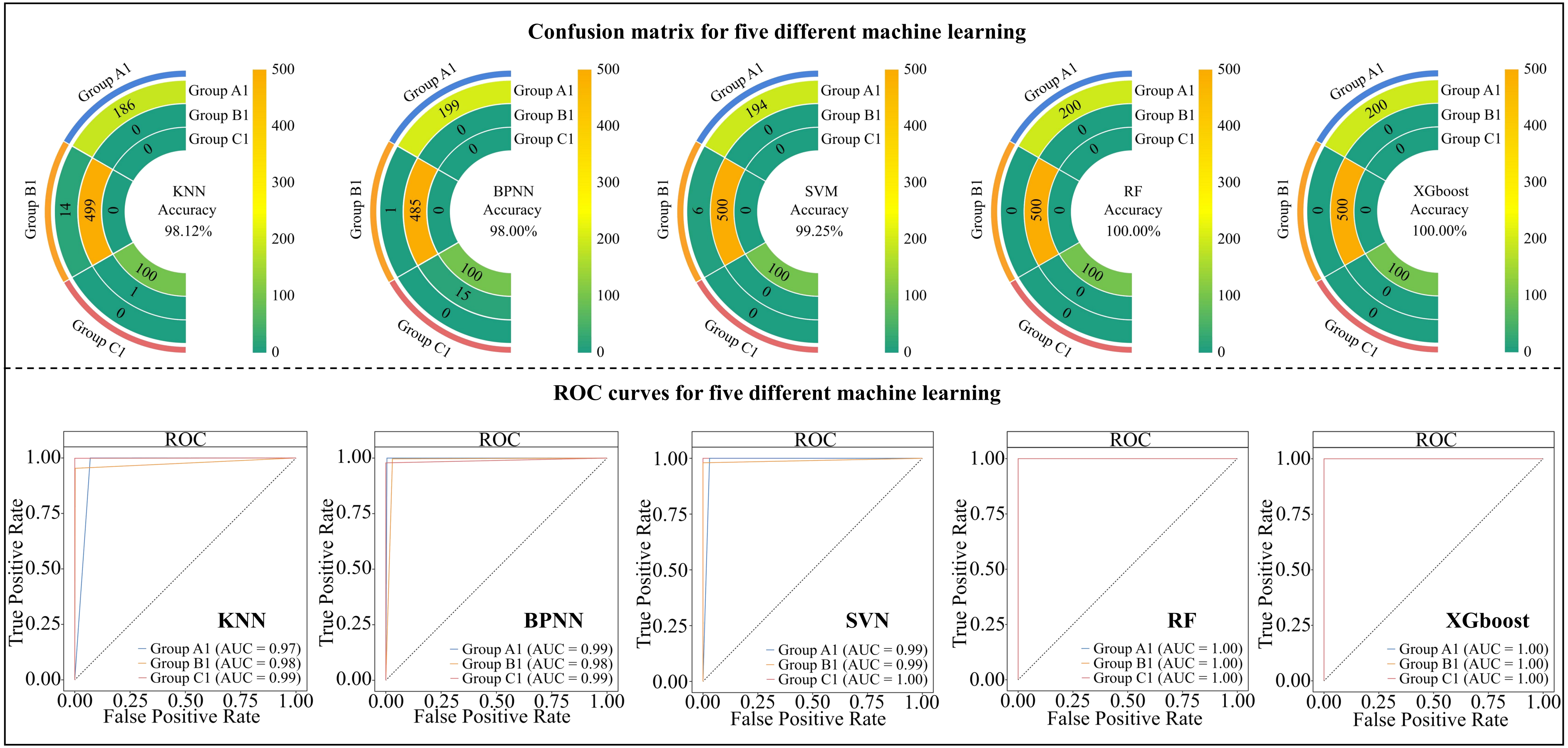

Figure S13. Machine deep learning based on available nitrogen, available phosphorus, and available potassium content of rhizosphere soil of 45 tea tree germplasm resources to validate classification accuracy. KNN, BPNN, SVN, RF and XGboost represent five machine deep learning such as k-nearest neighbor, back propagation neural network, support vector machine; random forest, eXtreme gradient boosting, respectively. Group A1, B1, C1 denote the three categories into which the 45 tea tree germplasm resources were classified after unsupervised K-means clustering based on the rhizosphere soil available nutrient contents of the 45 tea tree germplasm resources (see Supplementary Table S6 for the tea tree species corresponding to Group A1, B1, C1).

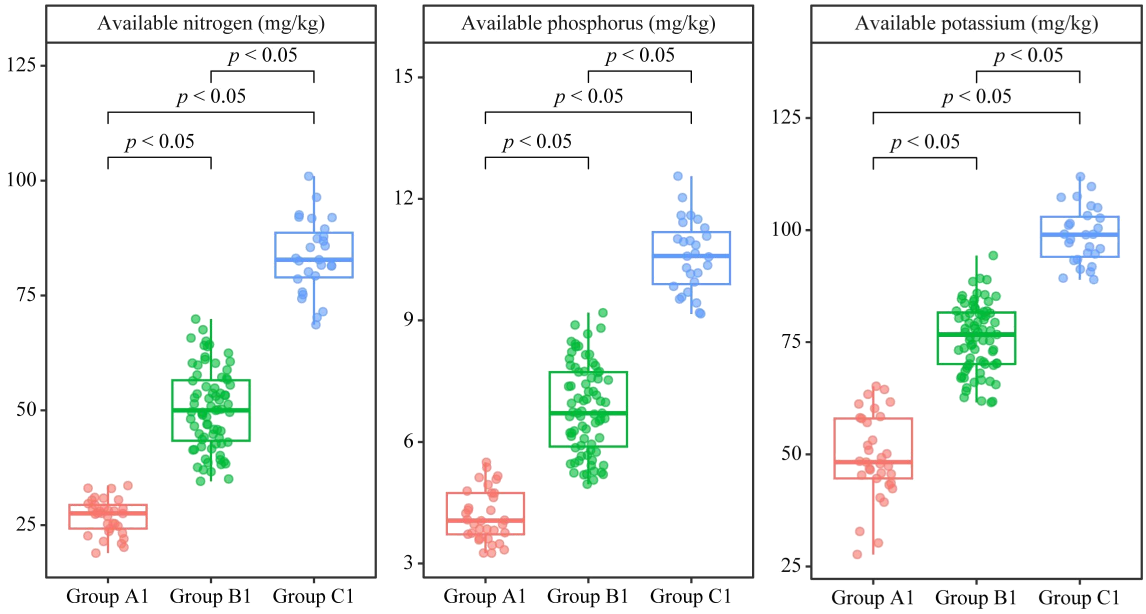

Figure S14. Difference analysis of available nitrogen, available phosphorus, and available potassium content of rhizosphere soil after 45 tea tree germplasm resources were classified into three groups. Group A1, B1, C1 denote the three categories into which the 45 tea tree germplasm resources were classified after unsupervised K-means clustering based on the rhizosphere soil available nutrient contents of the 45 tea tree germplasm resources (see Supplementary Table S6 for the tea tree varieties corresponding to Group A1, B1, C1). Wilcox.test was used to test the significance of differences between two groups at the $p < 0.05$ level.

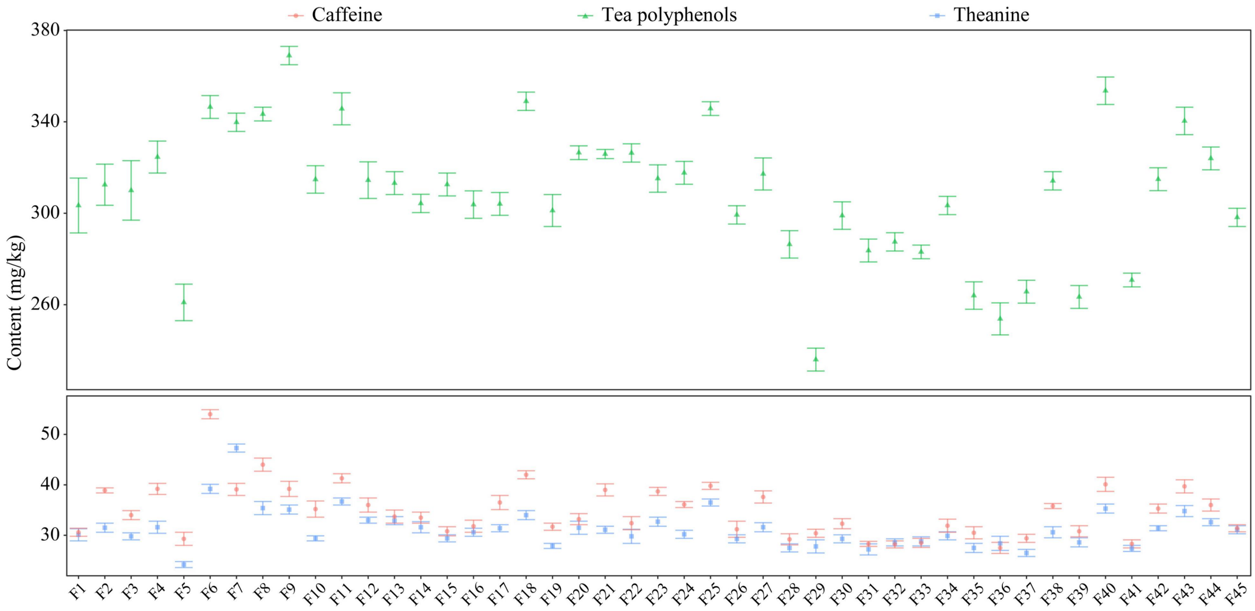

Figure S15. Content of tea polyphenols, theanine and caffeine in leaves of 45 tea germplasm resources. Data are presented as means and standard deviations (means ± SD).

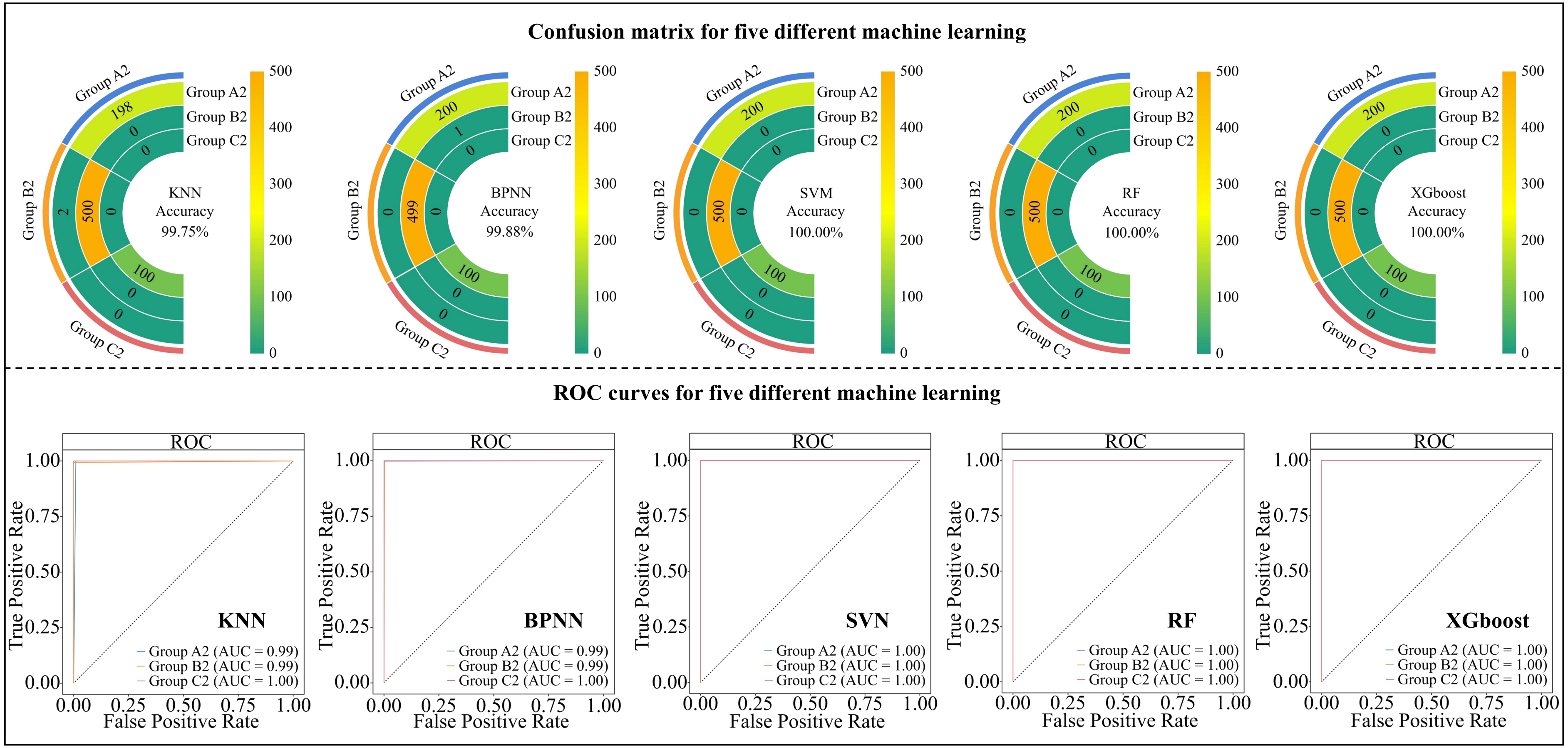

Figure S16. Machine deep learning based on the content of tea polyphenols, theanine, and caffeine in the leaves of 45 tea germplasm resources to verify the classification accuracy. KNN, BPNN, SVN, RF and XGboost represent five machine deep learning such as k-nearest neighbor, back propagation neural network, support vector machine; random forest, eXtreme gradient boosting, respectively. Group A2, B2, C2 denote the three categories into which the 45 tea germplasm resources were classified based on the content of leaf quality indexes of the 45 tea germplasm resources after unsupervised K-mean clustering (see Supplementary Table S7 for the corresponding tea varieties of Group A2, B2, C2).

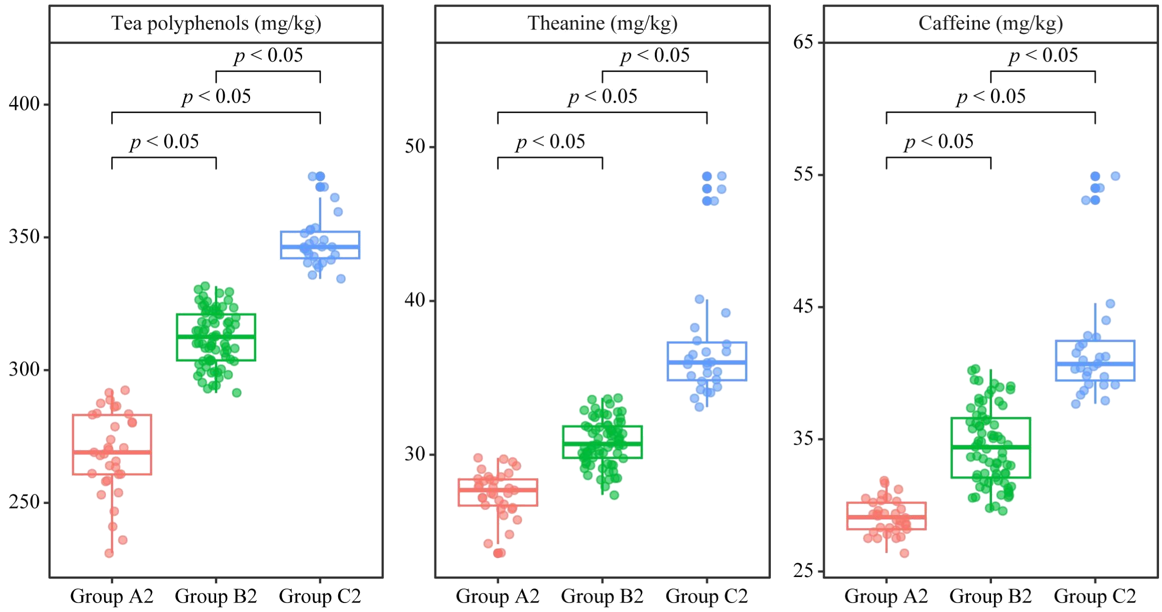

Figure S17. Analysis of differences in the content of tea polyphenols, theanine, and caffeine in leaves between groups after 45 tea germplasm resources were classified into three groups. Group A2, B2, C2 denote the three groups into which the 45 tea germplasm resources were classified based on the content of leaf quality indexes after unsupervised K-means clustering (see Supplementary Table S7 for the tea varieties corresponding to Group A2, B2, C2). Wilcox.test was used to test the significance of differences between two groups at the $p < 0.05$ level.

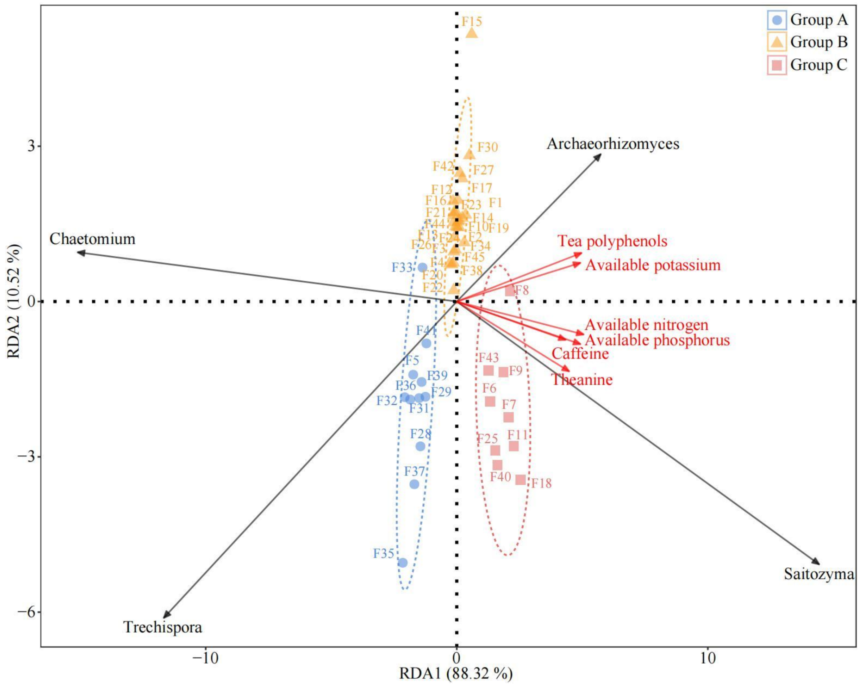

Figure S18. Redundancy analysis with quantitative data of 4 characteristic fungal genera, soil available nutrient content, and quality index content

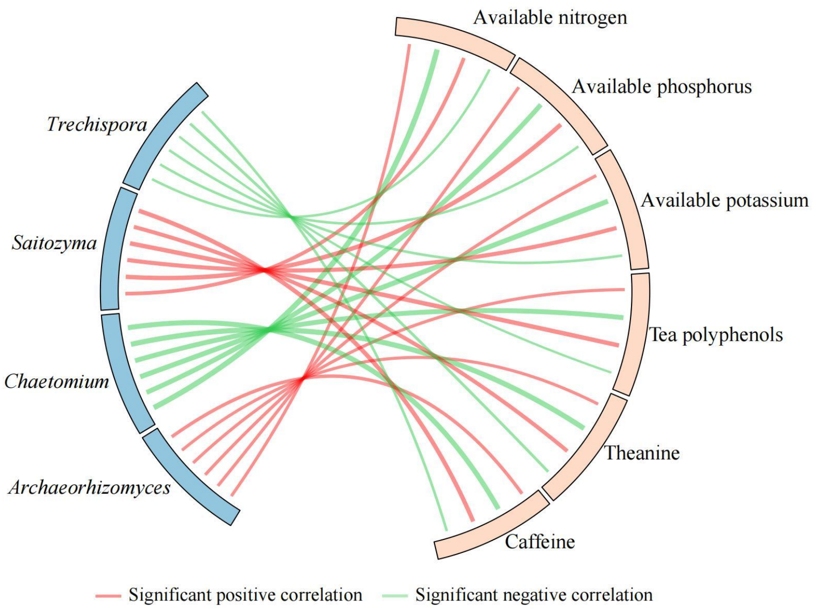

Figure S19. Correlation and interactions network analysis with quantitative data of 4 characteristic fungal genera, soil available nutrient content, and quality index content. Correlations were evaluated using Spearman's correlation coefficient, where $p < 0.05$ was considered a significant level of correlation.

Table S1. Numbers and names of 45 tea tree germplasm resources

No.	*Camellia sinensis* variety	No.	*Camellia sinensis* variety	No.	*Camellia sinensis* variety
**F1**	Baiyaqilan	**F16**	Shuixian	**F31**	Yanzhiliu
**F2**	Dahongpao	**F17**	Huangdan	**F32**	Tieluohan
**F3**	Yuqilin	**F18**	Beidou	**F33**	Queshe
**F4**	Aijiaowulong	**F19**	Baisuixiang	**F34**	Zimeigui
**F5**	Xiangtianmei	**F20**	Zihongpao	**F35**	Jinfenghuang
**F6**	Liulanxiang	**F21**	Bantianyao	**F36**	Foshou
**F7**	Jingui	**F22**	Meizhan	**F37**	Jinmaohou
**F8**	Zhengtaiyin	**F23**	Baijiguan	**F38**	Jinguazi
**F9**	Baimudan	**F24**	Huangguanyin	**F39**	Qiuxiang
**F10**	Zhuangyuanhong	**F25**	Jinmudan	**F40**	Baimaohou
**F11**	Xiaohongpao	**F26**	Lianyuanqiqu	**F41**	Ruanzhiwulong
**F12**	Laojunmei	**F27**	Qiqu	**F42**	Yanru
**F13**	Rougui	**F28**	Yusun	**F43**	Yelaixiang
**F14**	Huangmeigui	**F29**	Zhengtaiyan	**F44**	Jinyaoshi
**F15**	Chunlan	**F30**	Yujingliuxiang	**F45**	Jinmeigui

Table S2. Statistics of splicing results after sequencing of rhizosphere soil fungi of tea trees

**Sample ID**	**Raw tags (Mb)**	**Clean tags (Mb)**	**Clean/Raw tags ratio**	**Sample ID**	**Raw tags (Mb)**	**Clean tags (Mb)**	**Clean/Raw tags ratio**
F1	236,957	230,476	97.26%	F24	145,831	142,729	97.87%
F2	222,797	209,914	94.22%	F25	124,792	116,223	93.13%
F3	214,602	205,141	95.59%	F26	70,529	66,426	94.18%
F4	55,117	52,307	94.90%	F27	67,200	62,569	93.11%
F5	140,179	131,880	94.08%	F28	52,826	49,690	94.06%
F6	174746	168356	96.34%	F29	54,393	52,875	97.21%
F7	96,847	93,860	96.92%	F30	27,690	24,768	89.45%
F8	173,782	164,191	94.48%	F31	51,877	48,864	94.19%
F9	258,487	253,698	98.15%	F32	57,695	55,789	96.70%
F10	169,861	162,338	95.57%	F33	48,559	46,736	96.25%
F11	183,874	175,498	95.44%	F34	132,593	125,181	94.41%
F12	55,182	52,784	95.65%	F35	47,667	46,833	98.25%
F13	172,490	165,237	95.80%	F36	114,396	108,074	94.47%
F14	157,140	150,258	95.62%	F37	130,045	124,281	95.57%
F15	112,354	104,596	93.10%	F38	60,958	55,388	90.86%
F16	117,702	109,317	92.88%	F39	134,744	129,215	95.90%
F17	175,265	171,175	97.67%	F40	174,301	171,213	98.23%
F18	228,140	225,208	98.71%	F41	211,757	185,308	87.51%
F19	58,692	56,292	95.91%	F42	87,734	80,014	91.20%
F20	135,894	129,135	95.03%	F43	75,618	72,764	96.23%
F21	125,913	111,593	88.63%	F44	251,759	241,467	95.91%
F22	78,954	75,376	95.47%	F45	111,837	109,188	97.63%
F23	122,958	115,850	94.22%				
Total raw tags					5,702,734 Mb		
Total rlean tags					5,430,075 Mb		
Total Clean/Raw tags ratio					95.22%		

Note: F1~F45 represent the serial numbers of 45 tea germplasm resources (Supplementary Table S1); Raw tags are the spliced sequence obtained after filtering low-quality Fastq data; Clean tags are obtained after removing chimeras and short sequences from the spliced sequence.

Table S3. Distribution statistics of high-quality sequences after sequencing of rhizosphere soils of tea trees

Length (bp) distribute	Sequences reads
0-200	1,190,145
200-260	3,164,280
260-320	979,325
320-360	48,279
360-380	5,035
380-400	4,699
400-420	12,091
420-440	1,946
440-460	7,678
460-480	3,393
480-500	6,396
500-520	4,567
520-540	2,241
540-560	0
560-600	0

Table S4. Statistics on the number of OTUs in the rhizosphere soil samples of tea trees

Sample ID	Final tags	OTUs	Sample ID	Final tags	OTUs
F1	24,578	628	F24	24,578	653
F2	24,578	542	F25	24,578	665
F3	24,578	733	F26	24,578	602
F4	24,578	769	F27	24,578	594
F5	24,578	688	F28	24,578	638
F6	24,578	701	F29	24,578	707
F7	24,578	618	F30	24,578	592
F8	24,578	487	F31	24,578	682
F9	24,578	522	F32	24,578	658
F10	24,578	680	F33	24,578	658
F11	24,578	550	F34	24,578	631
F12	24,578	577	F35	24,578	472
F13	24,578	728	F36	24,578	733
F14	24,578	680	F37	24,578	733
F15	24,578	448	F38	24,578	716
F16	24,578	818	F39	24,578	741
F17	24,578	593	F40	24,578	549
F18	24,578	403	F41	24,578	497
F19	24,578	596	F42	24,578	495
F20	24,578	754	F43	24,578	717
F21	24,578	804	F44	24,578	641
F22	24,578	643	F45	24,578	544
F23	24,578	712			
Total OTUs			28,592		

Note: F1~F45 denote the serial numbers of 45 tea germplasm resources (Supplementary Table S1). Final tags is the number of tags corresponding to each sample in the final OTU table; OTUs is the number of OTUs finally obtained for each sample.

Table S5. K-means clustering by rhizosphere soil fungal abundance of 45 tea tree germplasm resources

Category	Sample No.	*Camellia sinensis* variety	Category	Sample No.	*Camellia sinensis* variety	Category	Sample No.	*Camellia sinensis* variety
Group A	F5	Xiangtianmei	Group B	F1	Baiyaqilan	Group C	F6	Liulanxiang
	F28	Yusun		F2	Dahongpao		F7	Jingui
	F29	Zhengtaiyan		F3	Yuqilin		F8	Zhengtaiyin
	F31	Yanzhiliu		F4	Aijiaowulong		F9	Baimudan
	F32	Tieluohan		F10	Zhuangyuanhong		F11	Xiaohongpao
	F33	Queshe		F12	Laojunmei		F18	Beidou
	F35	Jinfenghuang		F13	Rougui		F25	Jinmudan
	F36	Foshou		F14	Huangmeigui		F40	Baimaohou
	F37	Jinmaohou		F15	Chunlan		F43	Yelaixiang
	F39	Qiuxiang		F16	Shuixian			
	F41	Ruanzhiwulong		F17	Huangdan			
				F19	Baisuixiang			
				F20	Zihongpao			
				F21	Bantianyao			
				F22	Meizhan			
				F23	Baijiguan			
				F24	Huangguanyin			
				F26	Lianyuanqiqu			
				F27	Qiqu			
				F30	Yujingliuxiang			
				F34	Zimeigui			
				F38	Jinguazi			
				F42	Yanru			
				F44	Jinyaoshi			
				F45	Jinmeigui			

Table S6. K-means clustering of rhizosphere soil available nutrient contents with 45 tea tree germplasm resources

Category	Sample No.	*Camellia sinensis* variety	Category	Sample No.	*Camellia sinensis* variety	Category	Sample No.	*Camellia sinensis* variety
Group A1	F5	Xiangtianmei	Group B1	F1	Baiyaqilan	Group C1	F6	Liulanxiang
	F28	Yusun		F2	Dahongpao		F7	Jingui
	F29	Zhengtaiyan		F3	Yuqilin		F8	Zhengtaiyin
	F31	Yanzhiliu		F4	Aijiaowulong		F9	Baimudan
	F32	Tieluohan		F10	Zhuangyuanhong		F11	Xiaohongpao
	F33	Queshe		F12	Laojunmei		F18	Beidou
	F35	Jinfenghuang		F13	Rougui		F25	Jinmudan
	F36	Foshou		F14	Huangmeigui		F40	Baimaohou
	F37	Jinmaohou		F15	Chunlan		F43	Yelaixiang
	F39	Qiuxiang		F16	Shuixian			
	F41	Ruanzhiwulong		F17	Huangdan			
				F19	Baisuixiang			
				F20	Zihongpao			
				F21	Bantianyao			
				F22	Meizhan			
				F23	Baijiguan			
				F24	Huangguanyin			
				F26	Lianyuanqiqu			
				F27	Qiqu			
				F30	Yujingliuxiang			
				F34	Zimeigui			
				F38	Jinguazi			
				F42	Yanru			
				F44	Jinyaoshi			
				F45	Jinmeigui			

Table S7. K-means clustering of leaf quality index contents of 45 tea germplasm resources

Category	Sample No.	*Camellia sinensis* variety	Category	Sample No.	*Camellia sinensis* variety	Category	Sample No.	*Camellia sinensis* variety
Group A2	F5	Xiangtianmei	Group B2	F1	Baiyaqilan	Group C2	F6	Liulanxiang
	F28	Yusun		F2	Dahongpao		F7	Jingui
	F29	Zhengtaiyan		F3	Yuqilin		F8	Zhengtaiyin
	F31	Yanzhiliu		F4	Aijiaowulong		F9	Baimudan
	F32	Tieluohan		F10	Zhuangyuanhong		F11	Xiaohongpao
	F33	Queshe		F12	Laojunmei		F18	Beidou
	F35	Jinfenghuang		F13	Rougui		F25	Jinmudan
	F36	Foshou		F14	Huangmeigui		F40	Baimaohou
	F37	Jinmaohou		F15	Chunlan		F43	Yelaixiang
	F39	Qiuxiang		F16	Shuixian			
	F41	Ruanzhiwulong		F17	Huangdan			
				F19	Baisuixiang			
				F20	Zihongpao			
				F21	Bantianyao			
				F22	Meizhan			
				F23	Baijiguan			
				F24	Huangguanyin			
				F26	Lianyuanqiqu			
				F27	Qiqu			
				F30	Yujingliuxiang			
				F34	Zimeigui			
				F38	Jinguazi			
				F42	Yanru			
				F44	Jinyaoshi			
				F45	Jinmeigui			

Table S8. *q*RT-PCR primers for 20 characteristic fungal genera

No.	Microorganism	Forward primer	Reverse primer	Product (bp)
1	*Microdochium*	CTTTGAACGCACATTGCGCC	TCTCCCAACACTAAGCTAGGC	91
2	*Archaeorhizomyces*	CGATGAAGAACGCAGCGAAG	GCGCAATGTGCGTTCAAAGA	83
3	*Paraphaeosphaeria*	CTCGCCCCAAATTCATTGGC	AATGTTGCTTCGTGGATGCG	95
4	*Chaetomium*	AGGCCCTGAAAAGCAGTGG	GTGGTTTAACGGCCGGAAC	95
5	*Trichoderma*	CGGCCCCGAAATACAGTGG	CTCCCGGTGCGAGTTGTG	72
6	*Aspergillus*	AGAGTATACCTGTTTGAGCGTC	AGGGCCCAACACACAAGC	84
7	*Saccharomycopsis*	CGGATCTCTTGGTTCTCGCA	GTGCAATATGCGTTCAAAGATTCG	104
8	*Antrodiella*	AGCTGGAATGTTACCGAGCA	GCGGACGGTTAGAAGCTGAA	91
9	*Talaromyces*	AGGATCATTACCGAGTGCGG	CGCCAAAGCAACAGGTGTAT	73
10	*Passalora*	GCCTTAAAGTCTCCGGCTGA	GCCCGAACTCTTCAGCGATT	73
11	*Trechispora*	GTTGGATCTGGGGGCTCTG	TACAACGCCCAGTCGAGTTC	91
12	*Ramophialophora*	TTGCCGACCTAGTCTGAACC	TTGCAAACCGGACTTTCGGA	70
13	*Arcopilus*	AGGCCTCTCTGAGTCTTCTGT	GCATTTCGCTGCGTTCTTCA	91
14	*Fusarium*	AGCTTGGTGTTGGGAGCTG	TACGCTATGGAAGCTCGACG	75
15	*Mortierella*	AACGCAGCGAAATGCGATAC	GCGCAATATGCGTTCAAAGA	75
16	*Arthrobotrys*	CTTTGAACGCACATTGCGCC	TGAGGGGTTGTAATGACGCT	70
17	*Calcarisporiella*	TCGAATCTTTGAACGCACCT	TGTGTTGCTTTGCGATTCTC	75
18	*Purpureocillium*	GAACCAGCGGAGGGATCATT	CCGAGGCAACTGAGGTAAGG	73
19	*Diaporthe*	CTTTGAACGCACATTGCGCC	GCCAGGCTTGAGGGTTGAAA	77
20	*Saitozyma*	GCGGATAAAGGCAGGAGGAA	GCATTCCTCAGTCTCAGCCA	82
